# Harnessing microbial power to degrade hydrocarbon‐based plastics

**DOI:** 10.1002/mlf2.70088

**Published:** 2026-06-19

**Authors:** Hui Li, Xuanyu Tao, Lina Sun, Aifen Zhou, Jizhong Zhou

**Affiliations:** ^1^ Institute for Environmental Genomics University of Oklahoma Norman Oklahoma USA; ^2^ School of Biological Sciences University of Oklahoma Norman Oklahoma USA; ^3^ Research Centre of Ecology & Environment for Coastal Area and Deep Sea Southern Marine Science and Engineering Guangdong Laboratory (Guangzhou) Guangzhou China; ^4^ Department of Chemical Engineering Texas A&M University College Station Texas USA; ^5^ Earth and Environmental Sciences Lawrence Berkeley National Laboratory Berkeley California USA; ^6^ School of Civil Engineering and Environmental Sciences University of Oklahoma Norman Oklahoma USA; ^7^ School of Computer Sciences University of Oklahoma Norman Oklahoma USA

**Keywords:** environmental biodegradation, hydrocarbon‐based plastics, microbial consortia, plastic‐degrading microorganisms/enzymes

## Abstract

The growing global plastic waste crisis demands the development of urgent, effective, and sustainable solutions. While conventional recycling methods present intrinsic limitations, microbial biodegradation of plastic waste has emerged as a promising alternative. In this review, we explore the potential of using microorganisms to degrade major hydrocarbon‐based plastic polymers and discuss key aspects of this rapidly advancing field, including (i) isolation and characterization of novel microorganisms and enzymes in hydrocarbon‐based plastic biodegradation, (ii) development and streamlining of microbial consortia to improve hydrocarbon‐based plastic biodegradation efficiency, and (iii) investigation of natural biodegradation processes to illustrate the relationship between plastic degradation and environmental influence. We highlight practical biotechnological approaches and advanced computational tools in hydrocarbon‐based plastic degradation, as hydrocarbon‐based plastic represents the highest proportion of plastic waste while still lacking effective conversion strategies. Our ultimate goal is to integrate microbial biodegradation strategies into modern waste‐management systems and offer a feasible pathway toward a circular bioeconomy, one in which persistent plastic polymers are no longer treated as waste but are converted into renewable feedstocks that support sustainable resource recovery.

## INTRODUCTION

**Figure 1:**
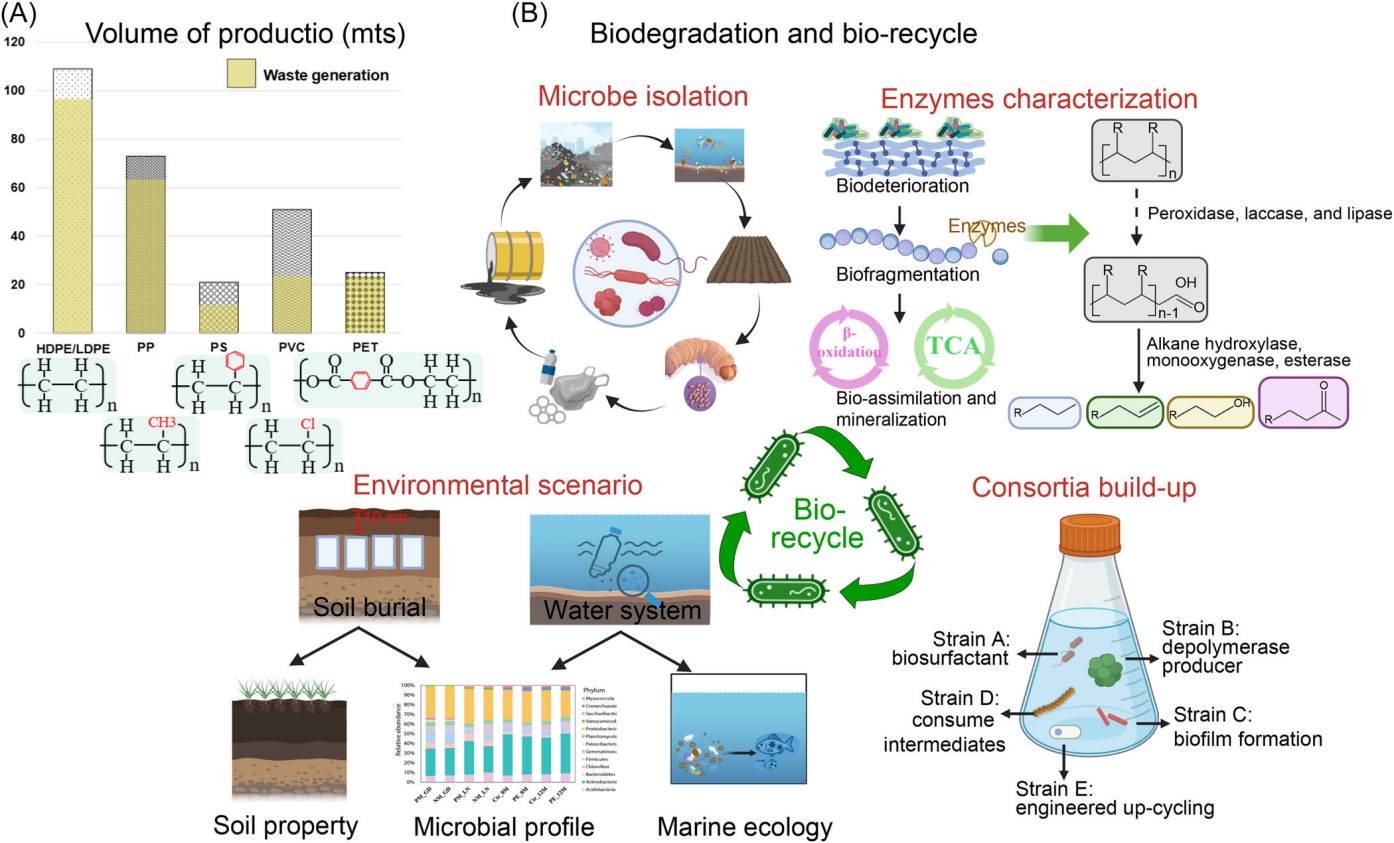
Plastic production, waste generation, and microbial biodegradation. (A) Annual production capacity^2^ and waste generation^3^ of different types of plastics. (B) Plastic biodegradation scope, encompassing microbe isolation from diverse environmental niches, enzyme characterization and functional profiling, consortium construction for enhanced degradation, and biodegradation strategies in environmental scenarios. HDPE, high‐density polyethylene; LDPE, low‐density polyethylene; PET, polyethylene terephthalate; PP, polypropylene; PS, polystyrene; PVC, polyvinyl chloride.

Plastics have become ubiquitous in modern society since their introduction in the early 20th century. Common petroleum‐derived plastics include polyethylene (PE), polypropylene (PP), polystyrene (PS), polyvinyl chloride (PVC), polyethylene terephthalate (PET), and polyurethane (PUR)^1^. Their versatility, low production costs, and range of properties (including lightweight and durability) have led to numerous applications, from food packaging and containers to construction materials, textiles, and automotive parts, although the durability that makes plastics so widely used also contributes significantly to long‐term environmental pollution. Figure 1A shows the annual production and waste generation of major types of plastics. PE has the highest production and nearly 90% waste generation, followed by PP. PVC is produced in large amounts but generates relatively less waste, whereas PET is produced in smaller amounts but most of it quickly enters the waste stream. It was estimated that roughly 7000 million tonnes (mts) of petroleum‐based plastics have been produced globally to date, and approximately 6300 mts of plastic waste have been released into and accumulated in urban environments, natural habitats, and marine ecosystems due to improper disposal and slow degradation rates^3^, ^4^. These accumulations result in visual pollution, harm to wildlife, and contamination of the food chain. As plastics break down, they form microplastics (MPs), that is, tiny particles (<5 mm) that pervade waterways and air, adsorbing toxic chemicals and posing risks to livestock, aquatic life, and human health through food consumption^5^, ^6^. A recent study reported that micro‐ and nanoplastic (<1 µm) concentrations in human tissues increased significantly from 2016 to 2024, with the highest accumulation consistently observed in the brain^7^.

Plastic waste can be managed through a combination of recycling, landfill disposal, and several degradation‐based processes. Landfill disposal, although still widely used, is the least preferred, as it leads to long‐term pollution in soil and aquatic ecosystems. Degradation‐based processes include photodegradation by ultraviolet (UV) exposure, thermal degradation by high temperatures, chemical degradation via oxidation and hydrolysis reactions, and biodegradation through microorganisms and their derived enzymes^8^. Among these processes, biodegradation offers a more environmentally friendly strategy, as it minimizes toxic gas emissions and energy use while enabling the conversion of plastic polymers into targeted value‐added products^9^.

Hydrocarbon‐based plastics like PE, PP, and PS are synthetic polymers that contain only carbon and hydrogen atoms; their strong C─C and C─H backbones make them highly resistant to microbial attachment and enzymatic degradation. In contrast, polymers such as PET contain hydrolyzable ester bonds and show lower hydrophobicity, making them more chemically labile and susceptible to enzymatic depolymerization. The intrinsic recalcitrance of hydrocarbon plastics to biodegradation varies with their molecular structures. PE is composed of simple ─CH₂─CH₂─ units, which confers high crystallinity and hydrophobicity, hindering microbial attack (Table 1), although its low UV resistance allows pretreatments to reduce crystallinity and enhance susceptibility to enzyme access. PP consists of a similar backbone to PE, with an additional methyl group on every other carbon, which increases its hydrophobicity and steric hinderance, and thereby reduces enzyme susceptibility. PS contains aromatic rings highly resistant to enzymatic cleavage, but its relatively low crystallinity and higher surface energy may offer more microbial attachment sites. Although PVC is not a true hydrocarbon plastic with the presence of chlorine atoms in its backbone, it is frequently grouped with PE, PP, and PS in biodegradation studies because of its high global production volume and the shared C─C backbone that contributes to comparable recalcitrance. The chlorine groups in PVC further restrict microbial degradation and may result in toxic byproducts, making PVC an essential component in plastic biodegradation studies^10^, ^11^, ^12^.

**Table 1:** Physicochemical properties of common types of plastics.

	Monomer	Density (g/ml)	Crystallinity (%)	Melting temperature (°C)	Surface energy (mN/m)/contact angle	UV stability
LDPE	─CH₂─CH₂─	0.91–0.93	30–50	102–115	~31.6/~96.0°	Fair
HDPE	─CH₂─CH₂─	0.95–0.97	70–90	125–137	~31.6/~96.0°	Poor
PP	─CH₂─CH(CH₃)─	0.85–0.94	30–50	135–175	~30.5/~102.1°	Fair
PS	─CH₂─CH(Ph)─	1.04–1.07	~0 (amorphous)	170–280	~34/~87.4°	Poor
PVC	─CH₂─CHCl─	1.16–1.55	0–10	100–260	~37.9/~85.6°	Fair‐good
PET	OH─CH₂─CH₂─OH, HOO─Ph─OOH	1.38–1.40	10–30	260–280	~39.0/~72.5°	Fair

HDPE, high‐density polyethylene; LDPE, low‐density polyethylene; PET, polyethylene terephthalate; PP, polypropylene; PS, polystyrene; PVC, polyvinyl chloride.

There are four essential steps in microbial biodegradation of plastics: bio‐deterioration, bio‐fragmentation, bio‐assimilation, and mineralization^13^. In the bio‐deterioration stage, microorganisms attach to and colonize the plastic surface and secrete enzymes that trigger oxidation reactions, thereby initiating the breakdown process. The surface chemical changes can be detected using Fourier transform infrared (FTIR) spectroscopy and nuclear magnetic resonance (NMR)^14^. During the subsequent bio‐fragmentation stage, polymer chains undergo hydrolysis and are cleaved into smaller molecules. These molecular weight and number (Mw and Mn) changes can be detected using gel permeation chromatography (GPC) or thermogravimetric analysis (TGA)^15^. Intermediate products such as alkanes and aldehydes can be quantified using gas chromatography‐mass spectrometry (GC‐MS)^16^. Following fragmentation, low‐molecular‐weight intermediates (<500 Mw) can then be assimilated by microorganisms and metabolized into CO_2_ and H_2_O. Increase in cell biomass and CO_2_ emissions are used as indicators of microbial activities and overall biodegradation efficiency^17^.

In this review, we explore established procedures and emerging techniques in hydrocarbon‐based plastic biodegradation, emphasizing their implications for biotechnology and prospective environmental influence. We first discuss how advances in the identification and engineering of novel microorganisms and enzymes have paved the way for more effective plastic degradation. Then, we analyze the importance of developing microbial consortia and upcycling strategies that both enhance degradation efficiency and enable the transformation of plastic waste into value‐added products. Finally, we consider the ecological impacts of plastic degradation and highlight how interactions between environmental factors and microbial activities shape the plastic degradation across diverse ecosystems (Figure 1B).

## DISCOVERY AND CHARACTERIZATION OF MICROORGANISMS IN PLASTIC DEGRADATION

**Figure 2:**
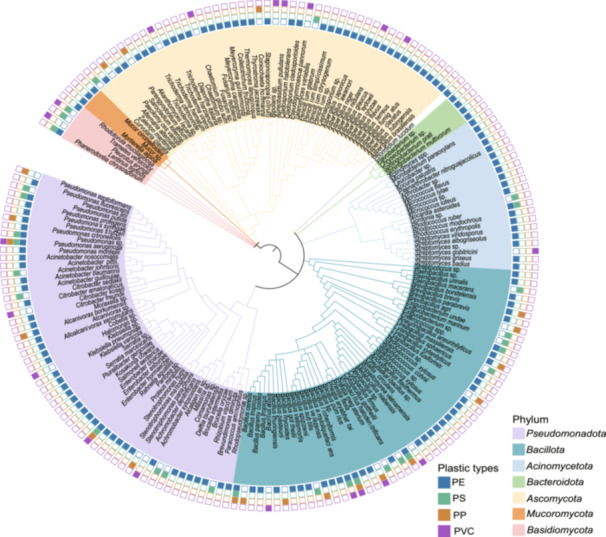
Phylogenetic tree displaying microorganisms identified with potential hydrocarbon‐based plastic‐degradation capabilities. The tree was constructed based on 16S rRNA alignment using ClustalW, with leaf colors representing respective phyla. Annotation rings outside the tree indicate the degradation potential of each microorganism for various hydrocarbon‐based plastics as specified in the key.

The studies investigating plastic biodegradation by microorganisms began shortly after synthetic plastics became a major environmental issue. By the 1990s, the research focused on the isolation of pure microbial strains with plastic‐degrading capabilities from diverse environments, including plastic‐contaminated soils, marine ecosystems, and the guts of plastic‐eating worms. Most of the single strains identified to date are bacteria, which can form biofilms on polymer surfaces or disrupt surface structures^18^ (Table 2). A comprehensive literature survey identified hydrocarbon‐based plastic‐degrading microorganisms spanning seven major phyla as of March 2026 (Figure 2). Bacteria, particularly those belonging to *Actinomycetota*, *Bacillota*, and *Pseudomonadota*, represent the majority of identified bacterial degraders.

**Table 2:** Bacterial and fungal species capable of degrading hydrocarbon‐based plastics.

Phylum	Microorganisms	Source	Polymer (additive, pretreatment)^a^	Experimental condition	Result	Reference
**Bacteria**						
*Actinomycetota*	*Rhodococcus ruber* C208	PE agricultural waste soil	LDPE film (no, untreated)	30°C for 8 w	0.86% per week WL	[19]
	*Rhodococcus ruber* C208	PE agricultural waste soil	PS flake (no, untreated)	35°C for 8 w	0.1% per week WL	[20]
	*Streptomyces* spp.	Nile River Delta	PE film (yes, heat)	30°C for 1 mo	NA	[21]
	*Micrococcus luteus*	Cow dung	HDPE (na, untreated)	37°C up to 90 d	3.85% WL	[22]
*Bacillota*	*Bacillus* sp. YP1, *Enterobacter asburiae* YT1	Gut of plastic‐eating waxworms	LLDPE film (no, untreated)	30°C for 60 d	10.7% and 6.1% WL	[23]
	*Bacillus cereus*, *Bacillus gottheilii*	Mangrove sediment	PP granules (no, UV)	33°C for 40 d	7.4%, 5.8% WL	[24]
	*Bacillus licheniformis*	Plastic waste dumping sites	PVC and PE film (no, untreated)	37°C for 30 d	15%, 32.2% WL	[25]
	*Bacillus thuringiensis* JNU01	Landfill	PE powder (na, chemical)	28°C for 30 d	NA	[26]
	*Exiguobacterium* sp. YT2	Gut of mealworm	PS film (yes, chemical)	Ambient for 60 d	7.4% WL	[27]
	*Priestia megaterium* S1	Gut of mealworm	LDPE, HDPE, PS beads (na, untreated)	28°C for 6 mo	36.1%, 31.9%, 28.6% WL	[28]
	*Brevibacillus borstelensis*	PE‐waste disposal site soil	LDPE (no, UV)	50°C for 30 d	11% WL, 30% Mw decrease	[29]
*Pseudomona‐dota*	*Pseudomonas* sp. AKS2	Municipal solid waste dumping ground soil	LDPE films (yes, untreated)	30°C for 45 d	5 ± 1% WL	[30]
	*Pseudomonas* spp.	Plastic dump yard	HIPS (yes, chemical)	30°C for 14 d	<10% WL	[31]
	*Pseudomonas aeruginosa*	Soil	PP powder (na, untreated)	30°C for 40 d	9.4% and 17.2% WL	[32]
	*Acinetobacter pitti*	Plastic waste soil	LDPE film (yes, UV)	30°C for 4 w	26.8% WL	[33]
	*Comamonas* sp.	Cerrado soil	UHMWPE powder (no, untreated)	28°C for 90 d	NA	[34]
	*Stenotrophomonas panacihumi* PA3‐2	Soil	PP powder (yes, untreated)	37°C for 90 d	Mw decrease	[35]
	*Alcanivorax borkumensis*	Mediterranean Sea	*Asperfillus clavatus* JASK1 + B22:F23	30°C for 80 d	3.5% WL	[36]
	*Oscillatoria subbrevis*	Domestic sewage water	LDPE strips (na, untreated)	25°C for 42 d	30% WL	[37]
	*Klebsiella* sp. EMBL‐1	Gut of armyworm	PVC film (yes, untreated)	30°C for 90 d	19.6% WL	[38]
	*Chelatococcus* sp. E1	Animal fodder compost	LMWPE (no, chemical)	58°C for 80 d	44.5% BD	[39]
**Fungi**						
*Ascomycota*	*Aspergillus clavatus* JASK1	Landfill soil	LDPE film (yes, untreated)	25–30°C for 90 d	35% WL	[40]
	*Aspergillus terreus* ATCC 20542		PP film (na, UV, and metallized)	30°C for 90 d	25.3% WL	[41]
	*Aspergillus flavus* PEDX3	Gut of wax moth	HDPE film (yes, untreated)	28 d	3.9% WL	[42]
	*Penicillium citrinum*	Municipal landfill soils	LDPE (na, untreated)	28°C for 90 d	38.8% WL	[43]
	*Penicillium variabile* CCF3219		PS film (no, ozonation)	24°C for 16 w	0.16% mineralization	[44]
	*Engyodontium album* MTP091	Plastic dump site	PP film (no, UV)	30°C for 12 mo	10% WL	[45]
	*Alternaria alternata* FB1	Marine	PE film (no, untreated)	25°C for 120 d	95% decrease Mw	[46]
	*Trichoderma viride* RH03	Dump site	LDPE film (yes, UV, autoclaved, and untreated)	3 mo	40%, 23%, and 13% WL	[47]
	*Cladosporium halotolerans*	*Galleria mellonella* larvae	HDPE powder (na, untreated)	25°C for 15 d	28% WL	[48]
	*Meyerozyma guilliermondii* ZJC1	Gut of waxworm	PE film (na, untreated)	60 d	13.9% WL	[49]
	*Phanerochaete chrysosporium*		PS membrane (yes, chemical)	30°C for 35 d	19.7% WL	[50]

BD, biodegradability; d, days; h, hours; HIPS, high‐impact polystyrene; LLDPE, linear low‐density polyethylene; LMWPE, low‐molecular‐weight polyethylene; mo, months; Mw, molecular weight; PE, polyethylene; UHMWPE, ultra‐high‐molecular‐weight polyethylene; UV, ultraviolet; w, weeks; WL, weight loss. ^a^The notation (additive, pretreatment) is defined as follows: “yes” indicates the presence of additives as specified in this paper, “no” indicates the confirmed absence of additives, and “na” signifies that no information regarding additives is provided.

Within *Actinomycetota*, the Gram‐positive aerobic bacterium *Rhodococcus ruber* C208 was observed to degrade low‐density polyethylene (LDPE) and PS at 0.86% and 0.1% per week, respectively^19^, ^20^. Members of the *Bacillota* phylum show particularly broad degradation potential. *Bacillus* sp. YP1, isolated from the gut of waxworms, degraded PE films at a rate of 1.34% per week^23^, while wetland isolates *Bacillus cereus* and *B. gottheilii* degraded PS at 1.30% and 1.01% per week, respectively^24^. *B. licheniformis* from plastic waste sites degraded PE by 7.53% and PVC by 3.50% per week^25^. Although these values appear to be promising, they are based on gravimetric weight‐loss assays, which are susceptible to artifacts (e.g., biomass adherence and chemical residues). Therefore, multiple complementary analyses were used to characterize plastic biodegradation. In the study of PE degradation by strain YP1, scanning electron microscopy (SEM) and atomic force microscopy (AFM) revealed pits and cavities of up to 0.4 µm in depth; X‐ray photoelectron spectroscopy (XPS) and attenuated total reflectance (ATR)‐FTIR detected new carbonyl (─C ═ O) groups' formation and increased O/C ratios. However, SEM and AFM cannot distinguish biological oxidation from physical abrasion, while FTIR and XPS require degraded components exceeding the 5%–20% detection limit to resolve signals from the strong, overlapping spectra of the bulk polymer matrix^51^. The *Pseudomonadota* phylum likewise includes efficient degraders. *Pseudomonas* sp. AKS2, isolated from soil, degraded LDPE by 5% in 45 days^30^, and an unclassified *Pseudomonas* strain from a plastic dump showed the ability to degrade high‐impact polystyrene (HIPS)^31^. In a HIPS degradation study, NMR was used to detect bromine release, high‐performance liquid chromatography (HPLC) was used for the identification of intermediates such as phenylethanol, and thermogravimetric analysis (TGA) was carried out for altered thermal stability detection. Two soil‐derived *Pseudomonas aeruginosa* strains degraded PP powder with weight losses of 9.35%–17.2% after 40 days^32^. In these experiments, water contact angle analysis showed substantial reductions in hydrophobicity (from 108° in controls to 75° and 57°), differential scanning calorimetry (DSC) revealed changes in crystallinity, and GC–MS identified degradation products such as C_29_ and C_35_ straight‐chain alkanes. Higher degradation efficiency of PVC, pretreated PP, and PE has been reported when *Pseudomonas* strains are co‐cultured with *Bacillus* species^52^, ^53^. It is important to note that clear reporting of pretreatment and plastic composition is necessary for interpreting DSC and TGA results, as these factors strongly influence thermal behavior. Instead, GC–MS and HPLC provide stronger chemical evidence of true chain cleavage by identifying depolymerization products and metabolic intermediates. Eventually, CO_2_ emission detection remains the most definitive signal of complete biodegradation, though it is technically challenging to measure due to the extremely slow degradation rates of plastics.

Insect gut microbiomes have also emerged as promising sources of plastic‐degrading bacteria. Different selection stringency levels have been used in enrichment strategies for isolating plastic‐degrading gut microorganisms. As in a study that isolated *Priestia* and *Exiguobacterium* from mealworms, gut mixture was first grown in nutrient‐rich broth containing plastics, and then on nutrient agar for pure colonies isolation^28^. A stricter method uses carbon‐free basal medium with plastics as the only carbon source, which creates stronger selection for degraders before plating on nutrient agar for isolation. *Enterobacter* from waxworms was isolated using this method^27^. The most stringent approach uses minimal salt medium with plastics as the sole carbon source, combined with repeated transfers to maintain selective pressure, as demonstrated in the isolation of *Klebsiella* from fall armyworm larvae^38^. Other bacterial degraders have been isolated from plastic‐contaminated soils, the Mediterranean Sea, and domestic wastewater systems^21^, ^33^, ^34^, ^35^, ^36^, ^37^. Additionally, thermophilic bacteria have shown accelerated degradation rates at higher temperatures. *Brevibacillus borstelensis* from soil achieved an 11% weight loss and a 30% reduction in LDPE molecular weight in 30 days at 50°C^29^, and *Chelatococcus* sp. E1 from compost degraded low‐molecular‐weight polyethylene (LMWPE) by 44.5% over 80 days at 58°C^39^. Thermophiles offer a key advantage in scalable biotechnology application because their enzymes remain stable and active under harsh industrial conditions.

While bacteria show a broader range of substrate utilization, fungi have demonstrated higher plastic‐degradation efficiency, with their hydrophobins facilitating adhesion to plastics' hydrophobic surfaces, and their powerful extracellular enzymatic system^54^. Fungal plastic degraders thus far predominantly belong to three phyla, *Ascomycota*, *Basidiomycota*, and *Mucoromycota*, which collectively account for 31% of identified degraders. Within *Ascomycota*, several *Aspergillus* species, including *A. clavatus* JASK1, *A. terreus*, and *A. flavus*, have demonstrated the ability to degrade PE and PP by forming biofilms and initiating oxidative processes^40^, ^41^, ^42^. In studies of *A. clavatus* JASK1, the Strum test detected 2.32 g/l of CO₂ after 4 weeks of LDPE culture. *Penicillium citrinum* isolated from plastic waste achieved a daily LDPE degradation rate of 0.43%^43^, while *P. variabile* mineralized 0.16% of ozonated PS film over 16 weeks at 24°C^44^. This study used ^14^C‐labeled PS to directly track mineralization through ^14^CO₂ production. *Engyodontium album* MTP091, isolated from a plastic dump site, showed enhanced PP degradation when co‐cultured with *Phanerochaete chrysosporium* NCIM 1170, a white‐rot fungus known for producing lignin‐degrading enzymes such as laccases and peroxidases^45^. Numerous additional fungal degraders have been isolated from marine environments^46^, plastic dumpsites^47^, and insect guts^49^. One notable example is a marine‐derived fungus *Alternaria alternata* FB1, whose transcriptome revealed 153 upregulated genes associated with plastic degradation. Heterologous overexpression of glutathione peroxidase and laccase in *Escherichia coli* confirmed enzymatic activity against PE films' degradation.

These studies have uncovered numerous plastic‐degrading microorganisms and analytical approaches; yet, they also reveal notable variability in experimental design and result interpretation. As listed in Table 2, different plastic physical forms, such as film, flakes, granules, and powders, which greatly differed in surface area and accessibility to microbial colonization, were used. Additionally, some studies do not specify the presence and composition of additives. Inevitably, when powders or flake forms are used, subsequent measurement of weight loss will be subjected to deviation from loss during washing steps. These variances significantly compromise the confidence and reproducibility of the experiment. As a result, despite significant individual advances, the field still needs a unified framework for benchmarking degradation efficiency and identifying broadly effective microbial or enzymatic strategies^55^, ^56^.

## DISCOVERY AND USE OF HYDROCARBON‐BASED PLASTIC‐DEGRADING ENZYMES

The 2010s marked a major shift in resolving the plastic biodegradation process from isolate‐based studies for identification of microorganisms with plastic‐degrading potential toward enzyme discovery, which reveals the specific catalytic steps and mechanisms driving the depolymerization procedure^57^. A defining example of this shift is the discovery of the bacterium *Ideonella sakaiensis*, which showed a major breakthrough in synthetic plastic degradation^58^. Further study revealed that this strain produces two enzymes, PETase and MHETase, that act sequentially to break down PET into its monomers^59^. Conclusively, enzyme‐based approaches offer greater flexibility than whole‐cell systems and can function across a wider range of temperatures, pH, and environmental conditions, which are more adaptable and scalable for plastic waste management^9^.

Extensive research has focused on enzymes that hydrolyze PET, though far fewer enzymes have been identified for degrading hydrocarbon‐based plastics, which lack hydrolyzable linkages and therefore require oxidative activation^60^. Therefore, enzyme discovery efforts have primarily focused on oxidative enzymes, such as lignin peroxidase, laccases, and monooxygenases (Table 3). Among ligninolytic enzymes, manganese peroxidase (MnP) from *P. chrysosporium* degraded the PE film after 8 days of incubation at 37°C^61^, while lignin peroxidase from the same species degraded up to 31% of PVC after 4 weeks at 25°C^62^. However, attributing PE degradation solely to MnP was not well supported, because degradation was observed only in the presence of Tween‐80 and Mn(II), conditions that are known to cause physical weakening or nonenzymatic oxidation of PE. A laccase from *R. ruber* C208 achieved a 2.5% LDPE degradation in 30 days^67^, and a laccase from *Trametes trogii* induced surface modification of PP after 4 months^68^. Within the monooxygenase group, the alkane hydroxylase AlkB from *Pseudomonas* sp. E4 degraded LMWPE at 19.3% over 80 days^69^, while AlkB from *P. aeruginosa* led to 19.6%–30.5% weight loss in LMWPE after 50 days at 37°C^70^. Although the expression of a single *alkB* gene was reported to confer LMWPE‐degrading capability to *E. coli*, the study did not provide sufficient methodological detail to confirm that the observed CO₂ production originated from PE degradation rather than from other medium components. Cytochrome P450 (CYP) monooxygenases have also shown plastic‐degrading potential: *E. coli* BL21 expressing a P450 from *Acinetobacter radioresistens* S13 was able to utilize medium‐ and long‐chain alkanes as the sole carbon source, indicating potential for hydrocarbon‐based plastic degradation^75^.

**Table 3:** Enzymes for biodegradation of hydrocarbon‐based plastics.

	Organism	Enzyme	Plastic type	Condition	Function	Reference
Ligninolytic enzymes	*Phanerochaete chrysosporium*	Manganese peroxidase	PE	37°C for 8 d	Mw decreased	[61]
	*Phanerochaete chrysosporium*	Lignin peroxidase	PVC	25°C for 4 w	31% WL	[62]
	Soybean	Soybean peroxidase	HDPE	60°C for 2 h	Hydrophilicity increased	[63]
	*Azotobacter beijerinckii*	Hydroquinone peroxidase	PS		Mw decreased	[64]
	*Alternaria alternata*	Glutathione peroxidase	PE	30°C for 48 h	Mw decreased	[45]
	*Trichoderma harzianum*	Laccase‐like oxidase	PE	Ambient for 15 d	0.5% and 0.6% WL	[47]
	*Psychrobacter* sp.	Laccase	PE	30°C for 7 d	13.2% WL	[65]
	*Lysinibaccillus fusiformis*	Laccase	PE	28°C for 8 w	New functional groups	[66]
	*Rhodococcus ruber*	Laccase	LDPE		2.5% WL	[67]
	*Trametes trogii*	Laccase	PP	28°C for 4 mo	Surface modification	[68]
Mono‐oxygenases	*Pseudomonas* sp.	Alkane hydroxylase	LMWPE	for 80 d	19.3% mineralized	[69]
	*Pseudomonas aeruginosa*	Alkane monooxygenase	LMWPE	37°C for 50 d	19.6%–30.5% WL	[70]
	*Acinetobacter johnsonii*	Alkane hydroxylase	LMWPS	37°C for 18 h	New functional groups	[71]
	*Acinetobacter radioresistens*	P450	Alkanes		Cell growth	[57]
	*Bacillus thuringiensis*	P450	PE	37°C for 18 h	Hydrophilicity	[26]
Other oxidase	Rice	Dioxigenase (HIS1)	PP		Surface modification	[72]
	*Galleria mellonella* larvae	Phenol oxidase (Demetra)	PE	90 min	2‐ketones detected	[73]
	Microplastic‐associated microbiota	Esterase	PS, HDPE, LDPE, and PVC	30°C for 30 d	6.94%, 8.71%, 7.47%, and 9.22% WL	[74]

LMWPS, low‐molecular‐weight polystyrene; min, minutes.

**Figure 3:**
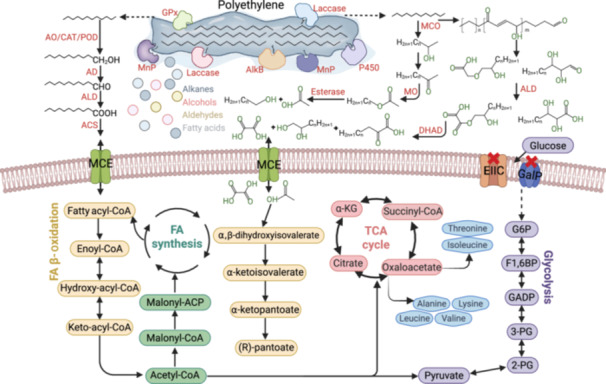
Conceptual model of PE degradation and downstream metabolic pathways in strain A34 grown on PE as the sole carbon source (adapted from Ref.^76^). As a representative example, we illustrate how integrated multi‐omics approaches can resolve the enzymatic and metabolic steps underlying microbial plastic degradation, thereby providing context for the plastic‐degradation pathways. Pathways were inferred from proteomics, FTIR, and GC–MS data. Solid arrows denote confirmed one‐step reactions, while dashed arrows indicate steps lacking proteomic support or involving unknown enzymes. The initial depolymerization of PE (dashed arrow) may involve multiple extracellular enzymes, including multicopper oxidase, catalase–peroxidase, esterase, and lipase. The orange frame highlights key extracellular depolymerization enzymes. Color coding: light brown, fatty acid β‐oxidation and associated biosynthetic pathways; light green, the TCA cycle, the pyruvate cycle, glyoxylate shunt, and the GABA pathway; light red, glycolysis, glycogenolysis, and the pentose phosphate pathway; and light blue, amino acid and secondary metabolite biosynthesis^76^. AD, alcohol dehydrogenase; FTIR, Fourier transform infrared spectroscopy; GABA, gamma‐aminobutyric acid; MCE, mammalian cell entry; PEP, phosphoenolpyruvate; TCA, tricarboxylic acid cycle.

So far, PE is the most extensively studied hydrocarbon‐based plastic in the context of biodegradation. A recent study of the *Rhodococcus* strain illustrated a comprehensive enzymatic pathway for PE biodegradation based on proteomics and multiple analytical results. Figure 3 adapted from previous research provides an integrated view of how bacteria convert recalcitrant PE into metabolically useful substrates that feed central carbon metabolism^76^. Extracellular PE‐depolymerizing enzymes, including multicopper oxidases, monooxygenases, and peroxidases, oxidized the C–C backbone, producing alkanes, alcohols, aldehydes, and fatty acids. The derivatives are imported into the cell via mammalian cell entry (MCE)‐family lipoprotein transporters and are involved in the β‐oxidation cycle, the tricarboxylic acid cycle (TCA) cycle, glyoxylate shunt, and downstream biosynthesis. Further functional study revealed that incubating PE with a recombinant enzyme mixture of a multicopper oxidase and a putative esterase led to surface functional group changes that neither enzyme alone could produce. This highlights the importance of enzyme synergy in the breakdown of resistant plastics like PE. Such synergistic action can be further exploited by integrating enzymes from different microbial resources. For example, the combination of PETase from *I. sakaiensis*, lipase B from *Candida antarctica*, and a scaffolding protein containing a carbohydrate‐binding module (CBM3) significantly enhanced PET catalytic conversion efficiency by 6.5‐ to 8‐fold^77^.

Metagenomics and metaproteomics have emerged as powerful approaches for uncovering the full enzymatic repertoire that microbial communities have evolved to degrade plastic in diverse environments^78^. For instance, metaproteomics analysis of a marine microbial consortium degrading an aromatic–aliphatic copolyester blend identified elevated levels of PETase‐like and MHETase‐like hydrolases^79^. To streamline the identification of plastic‐degradation enzymes and accelerate the functional screening of genes/enzymes associated with this process, a centralized database integrating currently available ones (Plastics‐Active Enzymes Database, PlasticDB, and PlasticEnz) is an essential prerequisite. Furthermore, *in silico* analysis is complemented by functional metagenomics, where environmental DNA libraries are expressed in model hosts (e.g., *E. coli*) and screened for plastic‐degrading activity, to facilitate the discovery of novel enzymes with high catalytic potential. One such example includes constructing and screening metagenomic libraries derived from microplastic‐associated microbiota for plastic‐degradation activity^74^, from which an esterase (CEstKAD01) with ≤55.94% similarity to known esterases/cutinases was selected and expressed in *E. coli*. The recombinant enzyme induced 6.94%, 8.71%, 7.47%, and 9.22% weight loss over 30 days in PS, high‐density polyethylene (HDPE), LDPE, and PVC, respectively. Even so, additional evidence, including direct demonstration of CEstKAD01–plastic binding, and the definitive characterization of the intermediates or oligomers produced are required.

## ENGINEERING STRATEGIES FOR IMPROVED HYDROCARBON‐BASED PLASTIC‐DEGRADATION EFFICIENCY

Recent advances in synthetic biology have enabled the engineering of microorganisms and enzymes to overcome the inefficiencies in their wild‐type counterparts. To enhance PET degradation capacity, multiple strategies including increasing enzyme thermal stability, modifying substrate binding cleft, and improving resistance to product inhibitors have been applied. For example, a structure‐guided protein‐engineering approach was used to enhance the activity of PETase from *I. sakaiensis* ^80^, where targeted mutations in the active site significantly improved its binding to and degradation of highly crystalline PET. Similarly, another study greatly enhanced the activity and thermostability of leaf–branch compost cutinase (LCC) by introducing a bisulfide bridge. This engineered variant functioned at 72°C and achieved up to 90% breakdown of PET bottles within 10 h^81^. Recently, increasing efforts have been made to use machine learning (ML) for the development of novel enzymes. For PETase engineering, ML pipelines include tools for enzyme classification, substrate and catalytic site identification, optimum condition evaluation, and enzyme activity prediction^82^, ^83^. By integrating simulation output with experimental results, ML methods are expected to accelerate enzyme engineering and even facilitate the creation of entirely novel enzymes^84^.

For hydrocarbon‐based plastics, research efforts have been focused on microbial engineering to improve plastic‐degrading efficiency. For example, an engineered *B. velezensis* strain expressing a synthetic PE‐degrading enzyme pathway (BCAv‐PEase) demonstrated an enhanced ability to break down PE microplastics^85^. This strain resulted in greater weight loss of PE films and significant changes in surface hydrophobicity, likely due to overexpression of the introduced enzymes. In addition to target enzymes for degradation, enhancing biofilm formation was also found to be an effective strategy, especially in aquatic conditions; biofilms can capture and concentrate microplastics, increase microbial attachment capacity, and shield microbial degraders from environmental stresses^86^. One modified *P. aeruginosa* Δ*wspF* strain overproduced biofilm, resulting in more than 90% capture efficiency of PVC microplastics and even causing floating plastics like PS to sink^87^. Beyond this, metabolic engineering further provides promising avenues for plastic upcycling. In one study, the intermediates produced from metal‐catalyzed depolymerization of mixed plastics (including PS, PE, and PET) were funneled into value‐added products such as β‐ketoadipate and polyhydroxyalkanoates (PHAs) by engineered *P. putida* ^88^. PHAs are a diverse family of bio‐based, biodegradable polymers that are gaining attention as sustainable alternatives to petroleum‐derived plastics. Upcycling synthetic plastics waste into value‐added, eco‐friendly biopolymers like PHAs provides a dual‐benefit solution that aligns with circular‐bioeconomy principles^89^.

At the enzyme level, artificial intelligence (AI)‐assisted discovery and ML‐guided design further accelerates the identification and implication of novel catalysts. For example, an AI‐powered tool called PEZy‐Miner, which integrates protein language models and binary classification, enriched known plastic‐degrading enzymes by up to 30‐fold and identified 27 high‐confidence candidates from a dataset of 100,000 sequences^90^. Another example is the application of a hidden Markov model (HMM)‐based sequence screening to reveal a novel dioxygenase (HIS1) from rice. Overexpression of the HIS1 gene confers *E. coli* with enhanced degradation capacity in various PP materials (e.g., microplastics and disposable products)^91^. Similarly, a thermophilic laccase in *Lysinibacillus fusiformis* (LfLAC3) was discovered through a combination of computational and activity‐based screening^72^. LfLAC3 showed robust activity in LDPE degradation, confirmed by the formation of oxidative functional groups and surface damage. Furthermore, combining modern tools with traditional laboratory techniques greatly accelerates the discovery and optimization of high‐performance enzymes. Recently, ancestral sequence reconstruction (ASR) was first exploited for the identification of nine resurrected ancient, generalist laccases, among which Anc52 showed the highest efficiency. Its catalytic activity was further enhanced by heat activation at neutral pH and site‐directed mutations at residues 203 and 288^66^. In laboratory techniques, directed evolution is also an effective approach for optimizing enzymes' performance by mimicking natural selection, allowing rapid improvement of catalytic traits without requiring detailed structural knowledge. One representative study started with force adaptation of a DNA‐shuffled laccase to extreme operational conditions in *Saccharomyces cerevisiae*, and then rationally redesigned protein regions based on computational modeling, finally overexpressed in *Aspergillus oryzae* with enhanced thermal and pH stability and higher polymer dye production^92^. This same strategy is applicable to plastic‐degrading enzymes, like laccases, monooxygenase, and peroxidase.

## DEVELOPMENT OF SYNTHETIC MICROBIAL CONSORTIA

In recent years, optimization of complex biological processes, such as plastic biodegradation, with the use of synthetic microbial consortia has shown rapid advancement. Unlike single‐strain systems, microbial consortia provide a division of labor structure, where each individual strain performs more efficiently through distributed metabolic functions. The development of microbial consortia for plastic degradation involves the deliberate assembly of multiple microbial strains with complementary physiological traits that synergistically break down plastic polymers, such as biofilm formation, surface attachment, enhanced enzyme secretion, cross‐feeding syntrophy, and stress resistance^93^. Consortium‐based strategies also unlock opportunities for upcycling plastic waste into value‐added products, such as organic acids, biosurfactants, and biopolymers, thereby contributing to a circular bioeconomy. To make more use of this, microbial consortia can be engineered for stability and functional resilience under dynamic environmental conditions, such as fluctuations in pH, temperature, salinity, or nutrient availability.

**Figure 4:**
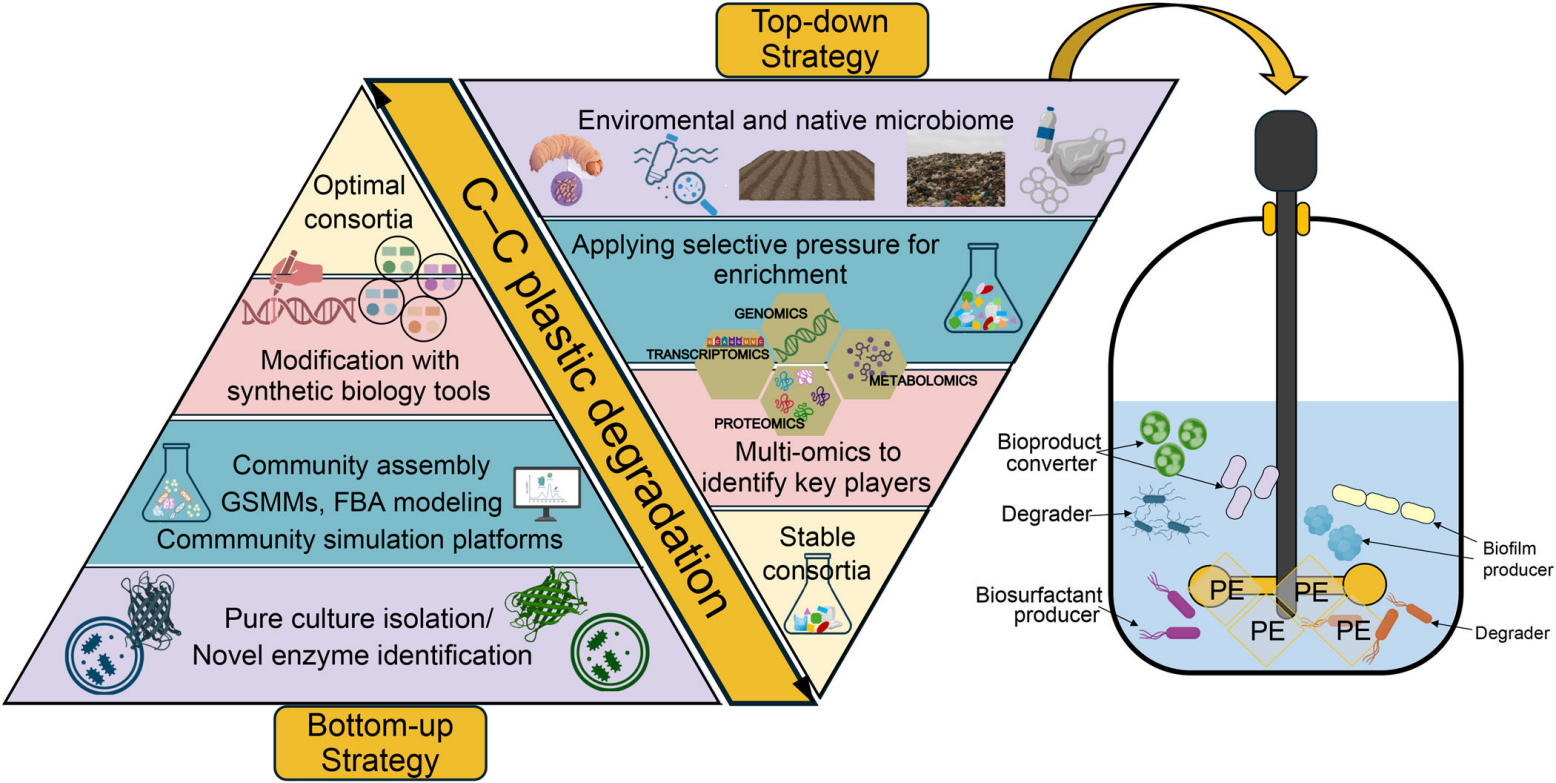
Framework for developing microbial consortia for plastic degradation using top‐down and bottom‐up strategies. The bottom‐up approach begins with isolating pure cultures and identifying novel enzymes, followed by use of GSMMs, FBA modeling, and community simulation platforms to refine strain selection, and predict cross‐feeding and competitive interactions. Genetic modifications using synthetic biology tools further optimize the consortia for enhanced degradation capacity. In the top‐down approach, environmental and native microbiomes are subjected to selective pressures to enrich plastic degraders. Multi‐omics analyses (genomics, transcriptomics, proteomics, and metabolomics) are then used to identify key microbial players, facilitating the formation of stable consortia for bioreactor applications. Both strategies converge in pilot‐scale applications, such as bioreactor systems, to upscale plastic degradation. FBA, flux balance analysis; GSMMs, genome‐scale metabolic models.

Two fundamental strategies that have been implemented for microbial consortia development are bottom‐up and top‐down approaches (Figure 4). In the bottom‐up approach, individual microbial strains or their engineered counterparts with well‐characterized functional capacities are assembled to accomplish complementary processes in plastic degradation and subsequent transformation into target products. The well‐defined function of these kinds of consortia allows fine‐tuned control over their metabolic pathways, although these artificially constructed consortia may lack ecological resilience and robustness necessary to confront environmental shifts and community disparity^94^. In contrast, the top‐down approach involves enrichment of microbial communities from natural samples such as landfill soils, marine ecosystems, and insect guts. Microbial communities are imposed with selective pressure by providing plastic as the sole carbon source, which allows enrichment of adapted and most efficient strains. This multi‐generation selection procedure offers opportunities to establish consortia with greater metabolic stability and ecological robustness. However, these kinds of consortia remain less controllable, as the exact interactions between species are not fully understood.

### Bottom‐up assembly of synthetic microbial consortia

A bottom‐up approach typically begins with the isolation and characterization of specific plastic‐degrading strains. A widely used strategy is to then combine the top‐performing degraders from the isolates and construct a consortium to achieve enhanced degradation efficiency. For example, a marine‐derived consortium containing four top degraders (*Vibrio parahaemolyticus*, *V. fluvialis*, *B. licheniformis*, and *Paenibacillus woosongensis*) achieved 47.07% ± 6.67% weight loss in LDPE samples over 120 days, which almost doubled that with the use of individual strains^95^. Similarly, a thermophilic consortium derived from sewage and landfill samples containing *Aneurinibacillus aneurinilyticus*, *Brevibacillus agri*, *Bacillus*. sp., and *B. brevis* led to weight losses of up to 58.2% for LDPE, 46.6% for HDPE, and 56.3% for PP over 140 days at 50°C^96^. Co‐culture of two waxworm‐gut isolates, *Meyerozyma guilliermondii* and *Serratia marcescens*, showed a significantly enhanced PE degradation rate of 15.87% at 30°C for 60 days^49^. Transcriptomic analyses indicated that *S. marcescens* upregulated multicopper oxidase, while *M. guilliermondii* activated TCA cycle genes in response to carbon limitation. However, combining top‐performing isolates does not always result in the highest degradation. While co‐culture of two PS degraders *Stenotrophomonas maltophilia* and *B. velezensis* resulted in 43.5% weight loss over 60 days at 30°C^97^, the addition of *A. radioresistens* reduced this rate to 17%. Although *A. radioresistens* achieved the highest PS degradation in monoculture, it functioned as a metabolic inhibitor rather than a synergistic partner. This highlights that metabolic synergy and ecological compatibility are critical in developing a successful consortium.

A major challenge in using complex microbial communities is the experimental variability resulting from largely undefined synergistic and antagonistic interactions among community members^98^. As traditional wet‐lab optimization can take months to years in trial and error, model‐guided design that integrates empirical data with computational modeling has therefore become an increasingly important strategy for community construction^99^. This approach typically begins with comprehensive strain characterization (genomic, proteomics, and phenotypic profiling), followed by computational analyses to guide consortium design. Genome‐scale metabolic models (GSMMs) decode genome sequencing data into a thorough list of organism‐specific metabolic reactions, while flux balance analysis (FBA) predicts the metabolic capacities and efficiency of individual strains under defined conditions. The strain‐level results can then be fed into community optimization models, such as dynamic multi‐species metabolic modeling (DMMM), and optimization‐based community modeling (OptCom)^100^. OptCom is specifically designed to predict metabolic interactions between community members, whereas DMMM upgraded FBA to a dynamic procedure that simulates real‐time changes over time. Together, these tools can guide the rational assembly of more functional microbial consortia by optimizing strain selection, identifying cross‐feeding and competitive interactions, and reducing metabolic redundancies. Examples from related fields illustrate the power of model‐guided design. Dynamic FBA has been applied to optimize inoculum ratios and aerobic‐to‐anaerobic phase transition of a consortium comprising a glucose uptake‐deficient *E. coli* strain and *S. cerevisiae*, which nearly doubled ethanol productivity compared with monocultures^101^. A flexible synthetic consortium optimization (FLYCOP) framework further advanced this modeling approach by systematically testing different initial parameters and optimizing final configurations. For example, FLYCOP was used in the simulation of a co‐culture of *Synechococcus elongatus* and *P. putida* for enhanced PHA production by exploring variations in sucrose export, nutrient limitations, and initial strain ratios^102^. Advances in these modeling approaches make them essential tools for designing and fine‐tuning bottom‐up microbial consortia^103^.

### Top‐down approach for synthetic microbial consortia development

The top‐down approach constructs microbial consortia by enriching naturally occurring communities with plastic‐degradation potential. Environmental samples including landfills, soils, plastic‐contaminated waterways, and insect guts are subjected to selective pressure by supplying plastic polymers as the sole carbon source. Serial enrichment facilitates the development of communities optimized for polymer degradation. Starting with a complex, pre‐existing microbial community, the approach to develop top‐down consortia is to direct the raw community toward a stable, highly efficient consortium for plastic degradation. In this process, several challenges need to be addressed. First, fast‐growing heterotrophs may outcompete the slow‐growing functionally important taxa during the enrichment process. Therefore, enrichment strategies need to be carefully designed to retain key functional strains. For example, a natural marine microbiome was enriched through long‐term acclimation using naturally weathered plastic films. Also, only microbes successfully colonized on the plastic surface were collected and used as a tailored biofilm consortium. In this tailored consortium, *Alcanivorax* and *Ochrobactrum* accounted for more than 40% of the relative abundance in these biofilms, with enrichment of additional known degraders such as *Bacillus* and *Pseudonocardia* ^104^. Also, in a long‐term experiment spanning 1150 days, PE and PP microplastics incubated in urban rivers showed significant selective enrichment of degrader communities. Throughout the incubation, community's diversity indices (Chao 1 and Invsimpson) reduced significantly, while PE and PP microplastics showed measurable degradation. Two distinct groups degraders became enriched, including core degraders consistently enriched across conditions, and “resuscitated” degraders constituting initially rare or dormant taxa became metabolically active under prolonged enrichment process^105^. Second, environmental‐sample‐derived consortia are often taxonomically and functionally complex; therefore, decoding of interactions among the complex community and tracking of dynamic changes at the taxonomic, strain, and functional gene levels are more necessary. In this regard, 16S rRNA amplicon sequencing and shotgun metagenomics are used to identify community shifts during enrichment processes; meanwhile, meta‐proteomics or meta‐transcriptomics are performed to elucidate functional pathways. For example, a 2‐year marine community incubation experiment revealed a progressive enrichment of genes associated with alkane degradation, including 21 metagenome‐assembled genomes (MAGs) encoding *alkB* and 23 MAGs encoding *CYP153*, primarily from *Proteobacteria* ^106^. These MAGs were also observed to encode genes in surfactant production and hydrocarbon transport. In another study, landfill soil enrichment culture achieved up to 55.6% LDPE weight loss over a 90‐day incubation, and the alpha diversity generally decreased during enrichment. Also, PICRUSt2‐based functional predictions revealed enhanced pathways associated with energy metabolism, stress responses, motility, and xenobiotic degradation^107^. In addition to microbe–microbe interaction, host–microbe interaction can also contribute to plastic biodegradation. In an insect‐associated system (mealworm), synergistic degradation mechanisms have been observed in which the host secretes high‐molecular‐weight emulsifiers to increase PS bioavailability, while gut microbes generate low‐molecular‐weight compounds to enhance respiratory activity on plastic surfaces^108^. Third, the stability and reproducibility of the enriched consortia need to be carefully evaluated for long‐term application. It has been shown that the enrichment outcomes strongly depend on the polymer type and incubation conditions. Studies of superworm gut microbiota have revealed distinct associations between plastic types and bacterial genera: PE with *Cronobacter*, PS with *Enterococcus*, PP with *Coprococcus*/*Morganella*, and PVC with *Proteus* ^109^. Therefore, careful control over enrichment parameters is essential for developing stable and reproducible consortia. A two‐stage enrichment method involving *in vivo* feeding with HDPE, PP, or PS, followed by *in vitro* incubation on the same polymers, resulted in stable, reproducible biofilm‐forming consortia^110^. To facilitate a comprehensive understanding and application of top‐down consortia, computational tools like MetaMIS in tracking community dynamics are very helpful. The platforms use temporal microbial community profiles from enrichment experiments to infer positive and negative interactions among community members, which allows to distinguish true functional degraders and helper taxa from opportunists, identifying low‐abundance but highly connected keystone microorganisms, and allows to evaluate whether the enriched community is likely to remain stable across transfers^111^. Overall, the use of top‐down enriched consortia has good potential, which depends on balancing selection conditions, resolving community interactions, and maintaining community stability across serial enrichment cycles.

## ENVIRONMENTAL INTERPLAY IN HYDROCARBON‐BASED PLASTIC DEGRADATION

Plastics enter terrestrial and aquatic ecosystems through multiple pathways, including agricultural use (e.g., mulch films, ~88% PE, 11% PP, and 1% biodegradable polymers), landfilling, direct littering, urban runoff, and wastewater discharge^112^. Once released into the environment, their degradation is regulated by a combination of abiotic and biotic processes that differ across ecosystems. In soils and waterways, abiotic factors such as UV radiation, temperature fluctuations, mechanical abrasion, and chemical oxidation break down plastics into smaller fragments, enhancing their susceptibility to microbial colonization^113^. Subsequent biotic degradation, driven by microbial and enzymatic activity, is also modulated by environmental conditions, including moisture, temperature, oxygen availability, pH, and nutrient levels^114^. In turn, plastics and their breakdown products can significantly alter ecosystem properties, influencing soil structure, microbial community dynamics, and key biogeochemical processes^115^.

### Plastic degradation in the terrestrial ecosystem

The accumulation and distribution of plastics and their fragmented forms—microplastics (MPs)—within soil matrices can profoundly alter physical, chemical, and biological properties, resulting in complex and wide‐ranging ecological impacts^116^. Mechanistically, plastics and MPs modify soil structure by physically occupying pore spaces and altering soil aggregation. Depending on their size, shape, and polymer type, plastics and MPs may block micropores, which reduces water flow and gas exchange, or creates additional macropores that affect soil aeration, decrease water retention, and modify wetting dynamics and evapotranspiration rates^116^. Plastics and MPs also alter soil chemistry and biological activity. They can directly affect nutrient pools or indirectly reshape nutrient cycling by modifying microbial enzymatic activities. For instance, soils amended with PVC MPs showed increased ammonium (NH₄⁺) concentrations and urease activity, coupled with reduced nitrate (NO₃⁻) levels and suppressed potential nitrification rates^117^. PVC MPs also induced clear shifts in microbial community composition, with temperature influencing the extent and direction of these changes^118^. Additionally, PVC exposure promoted the abundance of phthalate‐degrading microbes, which correlated with changes in urease activity and nitrogen cycling. Similarly, another study reported that high concentrations of PP MPs (28% w/w) significantly increased the activities of key soil enzymes^118^, including fluorescein diacetate hydrolase (FDAse) and phenol oxidase, and enhanced pools of dissolved organic carbon (DOC), dissolved organic nitrogen (DON), ammonium, nitrate, dissolved organic phosphorus, and phosphate.

The cumulative impacts of these physical, chemical, and biological disruptions profoundly affect plant–soil interactions and the biogeochemical processes that support ecosystem functioning^119^. Altered soil structure and nutrient cycling impair plant root development, reduce plant biomass, and diminish soil fertility and resilience^120^. MPs further stress plants by generating excessive reactive oxygen species (ROS), causing cellular damage, and disrupting nutrient uptake, especially magnesium and potassium, which are essential for chlorophyll synthesis^121^, ^122^. These physiological constraints reduce photosynthetic capacity and carbon fixation, thereby lowering rhizodeposition and ultimately decreasing the carbon inputs that fuel soil microbial activity. In addition, LDPE‐mulch has been shown to increase soil pH, reduce electrical conductivity (EC), and increase the C:N ratios, conditions that limit nutrient availability and promote microbial nitrogen immobilization, leading to lower plant‐available N and reduced biomass production^120^. Such feedback directly alters carbon and nitrogen cycling in planted soils.

**Figure 5:**
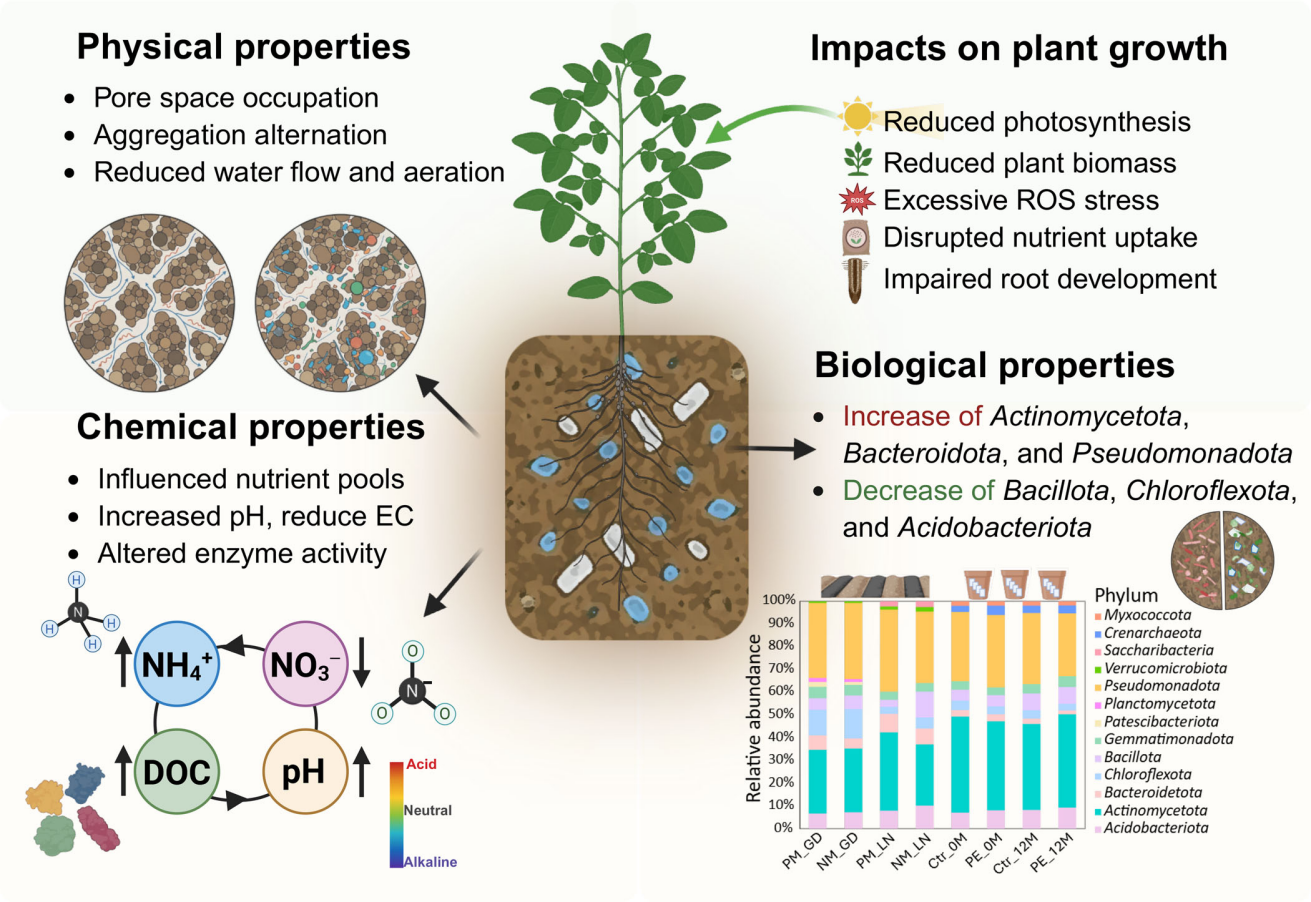
Effects of plastics and microplastics on soil properties and plant growth performance. Plastics and microplastics can alter soil physical structure^115^, influence chemical properties^116^, ^117^, and shift microbial communities^131^, ^132^. Together, these alterations can impair root development, reduce photosynthetic capacity, and ultimately suppress plant biomass production. EC, electrical conductivity; DOC, dissolved organic carbon; ROS, reactive oxygen species.

Because soil microbes regulate decomposition and nutrient transformations, changes in their composition or activity have immediate consequences for biogeochemical cycling^123^, ^124^, ^125^. Plastics and MPs impose selection pressures that subtly but persistently shift microbial community structure^126^. Several field studies report increased relative abundance of *Actinomycetota*, *Bacteroidota*, and *Pseudomonadota*, but decreased abundance of *Bacillota*, *Chloroflexota*, and *Acidobacteriota* in PE‐exposed soils^127^, ^128^, ^129^, ^130^, ^131^ (Figure 5). These trends have functional relevance: enriched taxa often harbor hydrocarbon‐degrading enzymes capable of metabolizing plastic‐derived carbon, whereas taxa that decline include oligotrophic decomposers associated with slower, soil organic matter (SOM)‐driven carbon turnover. Laboratory incubations corroborate these functional shifts. Early enrichment of fast‐growing heterotrophs during PE exposure is often followed by the dominance of *Actinomycetota* and *Pseudomonadota*—groups frequently associated with plastic oxidation and β‐oxidation pathways^133^, ^134^. Although responses vary across systems, these patterns indicate that plastic exposure can alter microbial succession and metabolic potential in ways that influence carbon degradation pathways. Context‐dependent responses further illustrate these dynamics. In a comparative incubation of forest soils and long‐term plastic‐contaminated soils, PE surfaces hosted distinct microbial successional trajectories, with plastic‐adapted soils showing early enrichment of known plastic degraders (e.g., *Rhodococcus*, *Streptomyces*, and *Nocardioides*) and earlier expression of PE‐degrading genes such as *fadA*, *fadJ*, and *alkM* ^135^. These differences demonstrate that historical exposure not only shapes the taxonomic composition of early colonizers but also primes the functional capacity of microbial communities, which may accelerate the onset of polymer oxidation and metabolic turnover of plastic‐derived substrates. Through these microbial‐driven changes, plastic degradation reshapes the soil environment in ways that regulate SOM decomposition, nitrification–denitrification pathways, and ultimately the retention or loss of carbon and nitrogen in plastic‐impacted soils.

### Plastic degradation in the marine and other waterway ecosystems

In aquatic environments, plastics and MPs provide stable surfaces that form distinct microhabitats called plastispheres, which facilitate microbial attachment, biofilm development, and prolonged colonization^132^. Studies consistently show that plastisphere communities differ markedly from those in surrounding water and often harbor genes involved in plastic degradation^136^. Because these biofilms concentrate organic pollutants, antibiotic resistance genes, and pathogens, they influence not only microbial ecology but also contaminant transport and biogeochemical fluxes^137^. Across marine systems, plastics support microbial assemblages that are compositionally and functionally distinct from planktonic communities. For instance, plastic debris in the North Atlantic harbored diatom‐rich biofilms and *Cyanobacteria* such as *Phormidium* and *Rivularia*, contrasting with *Prochlorococcus*‐dominated planktonic samples^138^. In the deep Southwest Atlantic, plastic substrates (HDPE, HDPE‐OXO, and PP) enriched families such as *Oleiphilaceae* and *Rhodobacteraceae*—taxa potentially associated with hydrocarbon oxidation^139^. Controlled incubations further support the role of MPs in selecting specialized degraders. Deep‐sea water exposed to PS MPs showed increases in *Alcanivoracaceae* abundance^140^, suggesting a potential role in plastic degradation, consistent with their known adaptation to hydrocarbon‐ and pollutant‐rich environments. Freshwater systems show similar trends. The microbial community composition (β‐diversity) on MPs is consistently distinct from those in surrounding water, sediments, or sand, indicating strong substrate specificity^141^. Besides, biodegradable (polylactic acid [PLA] + polybutylene adipate terephthalate [PBAT]) and non‐biodegradable (PE and PP) plastics were observed to support different successional trajectories, with non‐biodegradable plastics hosting more stable but less diverse communities^142^. Keystone taxa in nonbiodegradable groups emerged later compared to those associated with biodegradable plastics. Collectively, plastics act not merely as inert substrates but as active ecological niches that restructure microbial communities and alter small‐scale carbon and nutrient cycling in aquatic systems.

In contrast to soil plastispheres, which typically show lower diversity than bulk soil but stronger enrichment of plastic degraders, potential pathogens, and antibiotic‐resistance genes^143^, marine plastispheres, especially in stable open‐ocean waters, enrich halotolerant phototrophs, copiotrophs, and hydrocarbon degraders, with high dispersal and low environmental heterogeneity producing biofilms that are functionally specialized and network‐rich, showing greater complexity and more positive interactions than in freshwater^138^. Freshwater plastispheres form under sharper environmental gradient, shorter particle residence time, and strong watershed influence. Their communities are more locally sourced, spatially heterogeneous, and strongly filtered by nutrient pulses and redox fluctuations. Correspondingly, freshwater plastispheres show lower interaction‐network complexity, more modules, higher modularity, and greater competition than the surrounding water column^144^. Plastic‐degradation rates in marine and freshwater environments are strongly regulated by abiotic factors, including temperature, salinity, oxygen availability, pressure, light penetration, and nutrient availability, which regulate microbial metabolism. Salinity favors halotolerant degraders such as *Alcanivorax*, while freshwater systems select different functional groups^106^, ^145^. UV radiation enhances surface oxidation and increases microbial bioavailability^146^. Aerobic conditions promote oxidative depolymerization, whereas anoxic sediments or deep lakes suppress these pathways^147^. Recent estimates using the metric specific surface degradation rate (SSDR) indicate that HDPE degrades only 0–11 µm per year in marine environments, resulting in half‐lives of decades to centuries^148^.

## CURRENT CHALLENGES AND LIMITATIONS

In this review, we discuss discoveries of hydrocarbon‐based plastic‐degrading microorganisms from diverse environments, and their degradation enzymes and pathways, highlighting the growing recognition that plastic breakdown is neither rare nor taxonomically restricted but instead arises from a wide array of microbial strategies and enzymatic mechanisms. We also consolidate plastic‐degradation analytical approaches, consortia developments, and plastic degradation–environment interactions. Together, these insights set the stage for examining the remaining barriers that limit progress and the future directions that will guide the field toward scalable and impactful solutions.

Biodegradation presents a promising approach to reduce the impact of plastic pollution by harnessing the innate metabolic capabilities of microorganisms. However, leveraging microbial power for large‐scale plastic degradation, especially hydrocarbon‐based plastics, faces several key challenges that must be addressed. First, the plastic itself represents a major source of variation. Laboratory studies often use pure polymers, whereas most commercial plastics contain additives, such as stabilizers, plasticizers, and colorants, which can constitute up to 4.5% of the material^149^. These chemical compounds can hinder microbial colonization and disrupt enzymatic activity. Moreover, real waste streams typically contain multi‐forms and types of plastics (PE, PP, PS, and PET). At this stage, detailed reporting of experimental parameters, including plastic forms, additives, weathering history, plastic loading, and testing conditions (e.g., temperature, oxygen, light, pH, and duration), is essential for improving reproducibility and enabling meaningful comparison across studies. This is particularly important for studies conducted under environmental conditions, where abiotic factors such as sunlight‐driven photo‐oxidation, temperature fluctuations, and soil moisture profoundly alter plastic weathering, microbial colonization, and subsequent degradation kinetics. For instance, UV exposure can introduce oxygen‐containing functional groups that enhance microbial colonization^150^, ^151^. Soil moisture of ~50%–60% typically supports microbial activity, whereas oversaturation can cause anoxia and slows degradation^152^, ^153^. Pre‐aging processes, such as freeze–thaw cycles, further alter plastic properties and shape microbial responses and degradation potential^146^, ^154^.

Second, metrics used to assess plastic biodegradation also vary, ranging from gravimetric weight loss, surface erosion, or scratching, functional group changes, molecular‐weight reduction, intermediate product formation to isotope‐labeled CO_2_ detection. Each of these captures only specific stages of polymer breakdown. Surface roughening, cracking, or biofilm formation, for example, may indicate microbial colonization or abiotic activities, but are not definitive evidence of biodegradation. Conversely, isotope‐labeled carbon detection provides sufficient evidence for plastic assimilation, but is technically more challenging. Previous enzyme‐based plastic‐degradation research has also shown that inadequate negative controls and ambiguous analytical signals can lead to confounding interpretations or overestimation of enzyme activity^12^, ^38^, ^73^. Therefore, greater rigor in experimental validation and integration of complementary analytical methods are essential for distinguishing true biodegradation.

In addition, important mechanistic gaps remain in the understanding of hydrocarbon‐based plastic‐degradation pathways. Compared with PET degradation, where the discovery of *I. sakaiensis* and its PETase–MHETase system provided a clear enzymatic framework, the extracellular mechanisms responsible for initiating PE, PP, and PS breakdown remain much less defined. Identifying enzymes capable of attacking chemically inert C─C polymer backbones will likely require a comparable conceptual and mechanistic breakthrough.

Ultimately, a central challenge lies in translating laboratory discoveries into reliable, real‐world plastic biodegradation strategies. Although many plastic‐degrading microbes and enzymes have been reported under controlled flask conditions, their function can be highly varied under large‐scale processes. Microbial consortia are expected to accelerate degradation process and enable upcycling by combining complementary functions, but consortium development remains difficult. Enrichment from plastic‐contaminated environments is not always sufficiently selective, because fast‐growing non‐degrading taxa may outcompete slower but functionally important degraders^155^. Also, top‐down enrichment often results in consortia that contain unculturable members, making it challenging to resolve their interactions, assign functional roles, and ensure long‐term stability and reproducibility^93^.

## FUTURE PERSPECTIVES AND EMERGING OPPORTUNITIES

Despite substantial challenges, the field of microbial and enzymatic plastic degradation is entering a period of rapid advancement, propelled by breakthroughs in genomics, synthetic biology, and bioprocess engineering. In the coming decade, several research directions hold exceptional promise for transforming hydrocarbon plastic biodegradation from laboratory‐scale demonstrations into viable components of a circular bioeconomy.

One major opportunity lies in expanding the microbial repertoire capable of degrading diverse plastic polymers. Recent metagenomic analyses have uncovered plastic‐degradation‐associated genes across 12 microbial phyla—far more than the seven phyla from which plastic‐degrading microbes have been isolated^156^. This disparity highlights a substantial gap between the metabolic potential present in nature and the limited fraction of organisms that we are currently able to cultivate and characterize^156^. Many potential degraders may be slow‐growing, stress‐adapted, involved in syntrophic interactions, or tightly attached to plastic surfaces, making them difficult to recover with conventional methods. The iChip device, for example, has demonstrated that *in situ* diffusion‐based cultivation can recover previously uncultured microbes by allowing them to grow under natural environmental conditions^157^. Applying such strategies to underexplored habitats, including hot springs that may harbor thermostable enzymes, acidic drainage environments enriched in oxidative weathering enzymes, plastic‐rich sediments harboring microbes adapted to long‐chain polymers, and host‐associated microbiomes, could substantially expand the diversity of cultivable plastic degraders.

A second major opportunity involves high‐throughput functional screening, which has greatly accelerated the discovery of plastic‐degrading enzymes. Through metagenomics, metaproteomics, and functional assays, researchers have identified over 30,000 candidate enzymes from global soil and ocean metagenomes—nearly 60% of which are novel^158^. Soil microbiomes tend to show greater enzyme diversity, while marine microbial communities reflect adaptations to deep microplastic contamination. Notably, enzyme abundance correlates with environmental plastic levels, suggesting that microbial plastic‐degrading capacity is evolving in response to long‐term synthetic polymer exposure. As this genetic pool expands, an important direction will be capturing the active metabolic potential that bulk metagenomes may overlook. For example, the combination of subsurface environmental samplers with bioorthogonal non‐canonical amino acid tagging‐fluorescence activated cell sorting (BONCAT‐FACS)‐guided metagenomics has been used to identify translationally active hydrocarbon degraders and reveal key aerobic and anaerobic pathways that would not be detected through standard metagenomic sequencing alone^159^. Beyond single enzymes, multienzyme systems inspired by natural depolymerization complexes such as cellulosomes assemblies offer new opportunities for efficient plastic breakdown. For example, integration of PET‐degrading enzymes into the naturally scaffolded cellulosomes of *Clostridium thermocellum* enables whole‐cell PET biodegradation at 60°C, with engineered strains converting over 60% of a commercial PET film into soluble monomers within 14 days^160^. Adapting modular depolymerization complexes to perform sequential oxidation, breakdown, and metabolic funneling could provide powerful new routes for upcycling plastics like PE and PP into higher‐value products.

Moreover, design of synthetic microbial consortia through top‐down and bottom‐up approaches provides a powerful framework for developing next‐generation biodegradation systems, including those targeting hydrocarbon‐based plastics. In a recent study, researchers combined community‐scale metabolic modeling with ecological interaction analysis to identify key functional taxa within natural microbiomes and rationally reassemble them into streamlined, high‐performance consortia^94^. This top‐down strategy preserves beneficial interactions from complex communities, while bottom‐up design enables the selection and engineering of specific metabolic pathways needed for efficient pollutant breakdown. Such integrative approaches can be readily extended to hydrocarbon‐based plastic degradation, where cooperative division of labor is essential. Achieving this requires high‐throughput tools for screening, engineering, evolving, and evaluating complex microbial communities. Computational modeling offers critical support in predicting interactions and outcomes, though current models are often limited by oversimplified assumptions, incomplete annotations, and difficulty in capturing real‐time ecological dynamics. Iterative refinement through experimental validation and high‐quality multi‐omics data will therefore be critical for engineering consortia that remain stable and functional as complexity increases.

Looking ahead, it is realistic to envision the development of integrated, experimentally validated pilot‐scale frameworks for advancing both synthetic microbial consortia and hybrid biological–chemical recycling systems over the next decade. This time frame is supported by the rapid progress already achieved in adjacent fields such as wastewater treatment, anaerobic digestion, lignocellulosic bioconversion, and enzymatic PET recycling, where complex consortia and solid–liquid bioreactors are routinely operated at industrial scales. Building on these precedents, a practical roadmap for plastics includes (i) constructing, modeling, and adaptively evolving microbial consortia tailored to specific waste streams—such as mixed PE/PP films, multilayer packaging, and PS‐rich fractions—to accommodate the heterogeneity of real‐world feedstocks; (ii) developing and optimizing solid–liquid and slurry bioreactors capable of quasi‐continuous operation on pretreated polymer substrates, with strategies for enhancing mass transfer, regulating pH and redox conditions, and maintaining community stability; (iii) benchmarking enzymatically generated intermediates (e.g., oxidized oligomers and short‐chain hydrocarbons) as standardized inputs for catalytic upcycling units to enable efficient hybrid biological–chemical conversion pathways; and (iv) integrating these coupled systems with rigorous techno‐economic (TEA) and life‐cycle assessment (LCA) to identify conditions under which microbial–chemical integration becomes environmentally advantageous and economically competitive with mechanical or purely chemical recycling. Together, these steps outline an ambitious, yet feasible pathway for transforming current proof‐of‐concept biodegradation strategies into deployable technologies that can contribute meaningfully to a circular bioeconomy.
